# Mobile Health App for Japanese Adult Patients With Asthma: Clinical Observational Study

**DOI:** 10.2196/19006

**Published:** 2020-08-14

**Authors:** Norihiro Harada, Sonoko Harada, Jun Ito, Ryo Atsuta, Satoshi Hori, Kazuhisa Takahashi

**Affiliations:** 1 Department of Respiratory Medicine Juntendo University Faculty of Medicine and Graduate School of Medicine Tokyo Japan; 2 Research Institute for Diseases of Old Ages Juntendo University Faculty of Medicine and Graduate School of Medicine Tokyo Japan; 3 Atopy (Allergy) Research Center Juntendo University Faculty of Medicine and Graduate School of Medicine Tokyo Japan; 4 Department of Electric Medical Intelligence Management Juntendo University Faculty of Medicine and Graduate School of Medicine Tokyo Japan

**Keywords:** asthma, cough variant asthma, mobile health, ResearchKit

## Abstract

**Background:**

Inappropriate asthma control reduces quality of life and causes increased exacerbations. Mobile health (mHealth) employs information and communication technology for surveying health-related issues.

**Objective:**

This noninterventional, observational study assessed current real-world asthma control levels among Japanese patients with asthma and cough variant asthma (CVA) using the Zensoku-Log app.

**Methods:**

We developed the app using the ResearchKit platform and conducted a mobile-based, self-reporting, observational survey among patients with asthma and CVA. The app was downloaded 7855 times between February 2016 and February 2018, and enabled collection of data on symptoms, comorbidities, quality of life, medications, asthma control, and adherence.

**Results:**

Of the 1744 eligible participants (median age 33 years; range 20-74 years; male-to-female ratio 38.7:61.3), 50.97% (889/1744) reported unscheduled visits, 62.84% (1096/1744) reported regularly scheduled visits, 23.14% (402/1737) smoked, and 40.75% (705/1730) had pets. In addition, 91.89% (1598/1739) of participants had atopic predisposition, including allergic rhinitis and atopic dermatitis. Daily inhaled corticosteroid and oral corticosteroid treatment had been prescribed for 89.45% (1552/1735) and 22.07% (383/1735) of participants, respectively. Although an asthma control questionnaire demonstrated poor asthma control in 58.48% (1010/1727), a leukotriene receptor antagonist, theophylline, and a long-acting muscarinic antagonist had been prescribed for only 30.66% (532/1735), 15.91% (276/1735), and 4.38% (76/1735), respectively. The Adherence Starts with Knowledge 12 total score was 29. In the 421 participants who repeated the questionnaire, asthma control increased significantly between the initial and last rounds (*P*=.002).

**Conclusions:**

Users of this mHealth app in Japan had poorly controlled asthma and may need more treatment for asthma and their comorbidities. Repeated app users demonstrated improved asthma control.

**Trial Registration:**

UMIN Clinical Trial Registry UMIN000021043; https://upload.umin.ac.jp/cgi-open-bin/ctr_e/ctr_view.cgi?recptno=R000023913.

## Introduction

### Asthma

Asthma is one of the most common chronic diseases that poses a serious global health problem; it affects around 334 million people worldwide and its prevalence is increasing every year [1]. In Japanese adults, asthma prevalence has increased from about 1% to 6%-10% since the 1960s [2]. Although regular follow-up is recommended for patients with asthma, adherence to asthma treatment guidelines is generally poor in many countries [3-6]. As a result of poor adherence, appropriate asthma control is not achieved, which contributes to reduced quality of life and depressive symptoms, and causes sleeplessness, daytime fatigue, and school and work absenteeism as well as an increased number of exacerbations [7-9]. Previous reports have shown that adherence to asthma medication regimens tends to be not so good in Japanese patients with asthma, even though they are under specialist care for their asthma [10-13]. These data suggest that for many Japanese patients, asthma remains poorly controlled, despite the availability of nationally promoted standards and improved agents for asthma treatment.

### Mobile Health App

Mobile health (mHealth) involves the use of information and communication technology to improve the understanding of health care and is superseding previous forms of surveys, including traditional postal mail, telephone, and web page–based surveys [14,15]. Smartphones, which are used by around 40%-60% people in Japan, are used to collect real-world data directly from patients, which has been shown to be both cost-effective and fast [16-19]. mHealth research apps are often based on the ResearchKit platform (Apple Inc.), an iOS-based open-source framework for mobile medical research that was released in 2015. Moreover, iPhones (Apple Inc.) are used by around 60% of Japanese smartphone users, based on 2016 figures [20]. ResearchKit has been used in observational real-world studies of cardiovascular health, asthma, rheumatoid arthritis, Parkinson disease, type 2 diabetes, and cancer [14,17-19,21-23]. Although ResearchKit-based apps have been used in real-world observational studies of asthma, there are currently no studies using this app for Japanese patients with asthma. This noninterventional and observational study using the Zensoku-Log app assessed the current levels of asthma control as reported by Japanese patients with asthma and cough variant asthma (CVA) in a real-world setting.

## Methods

### App and Participant Recruitment

We created and developed the Zensoku-Log app using the ResearchKit platform of Medical Logue, Inc. The Zensoku-Log app was released on February 16, 2016, through the Apple App Store in Japan for iPhone 6, iPhone 6 Plus, iPhone 6s, and iPhone 6s Plus (Apple Inc.). Upon downloading and opening the app, participants had to view all informed consent screens and read about the risks and benefits of participating in the study and their right to withdraw from participation. During the electronic informed consent process, participants agreed to share data with the understanding that their personally identifiable information would be collected; they were also provided with an option for *Consent withdrawal* in the app that will allow them to withdraw from the study at any time without having to provide reasons. Once they consented to participate, they answered questions related to eligibility screening (age 20 years or older with physician-diagnosed asthma or CVA) and questions to confirm the certainty of their diagnosis of asthma or CVA (Multimedia Appendix 1).

This study was reviewed and approved by the Juntendo University Research Ethics Committee (Tokyo, Japan), and was registered in the UMIN Clinical Trial Registry (UMIN000021043) on February 16, 2016 (http://www.umin.ac.jp/). The app recorded all data collected for this study through a set of web services for data storage and security developed and operated by C2, Inc.

**Figure 1:**
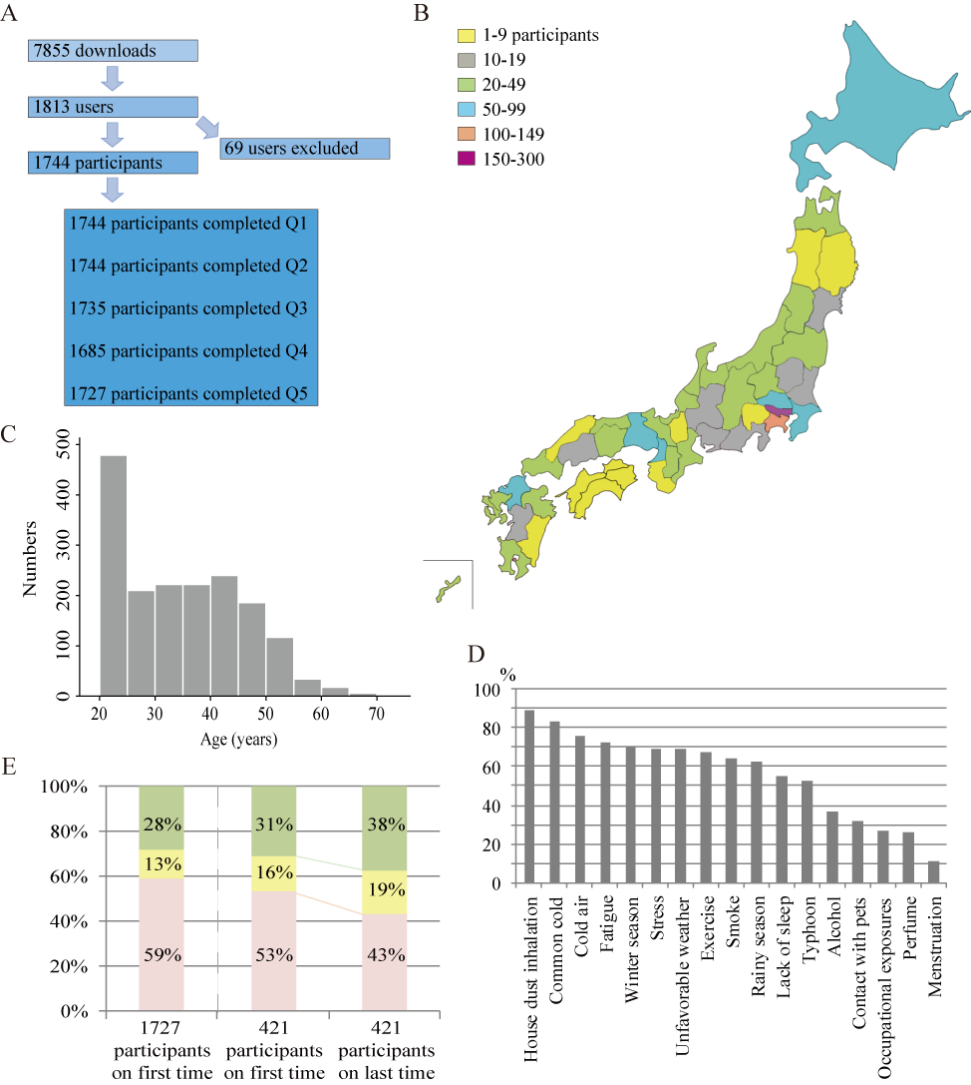
Recruitment process, geographic distribution, asthma triggers, and monthly Mobile Asthma Control Questionnaire (MACQ) surveys. (A) Patient recruitment process. (B) The geographic distribution of baseline users in Japan. (C) The age distribution of baseline users. (D) Percentages of factors that worsen asthma. (E) Proportion of patients with asthma control based on the MACQ surveys. The left column indicates the MACQ score for the 1727 patients who initially answered. Among the 421 patients who repeated the MACQ survey, the middle and the right column represents the MACQ score for the initial round and the last round, respectively. Green, yellow, and pink columns indicate total control (score of 15), well control (score of 13 or 14), and poor control (score of less than 13) of asthma, respectively.

We conducted this mobile-based, self-reporting, observational survey of patients with asthma in Japan between February 2016 and February 2018. The Zensoku-Log app was downloaded 6491 times within 2 weeks of its launch and was downloaded 7855 times overall during the study period. Of the users who downloaded the app, 1813 consented to participate in the study and agreed to share their data; however, 69 users were excluded (17 had input incorrect information and 52 were not diagnosed as having asthma or CVA). The remaining 1744 participants completed the Question 1 (Q1) and Question 2 (Q2) sections of the survey questionnaire (Figure 1A and Multimedia Appendix 1).

### Study Survey Design

We were unable to use certain standard validated questionnaires and quality of life surveys for patients with asthma directly in this study due to licensing constraints. Therefore, our asthma specialists developed a survey questionnaire by incorporating the general content used by validated survey instruments. Our survey questionnaire evaluated characteristics, including medical history, complications, familial atopic predisposition, social history, asthma trigger, and symptoms, in Q1 and Q2, asthma medications in the Question 3 (Q3) section, adherence to asthma medication in the Question 4 (Q4) section, and asthma control in the Question 5 (Q5) section (Multimedia Appendix 1). After the included participants (N=1744) initially answered Q1, 1744, 1735, 1685, and 1727 participants completed Q2, Q3, Q4, and Q5, respectively (Figure 1A). Furthermore, the Zensoku-Log app could collect user locations (latitude/longitude) and atmospheric pressure optionally at any time using the device’s global positioning system. After the first survey, users were able to repeat their answers in Q4 and Q5 optionally.

### Monthly Questionnaire for Assessing Asthma Control

The monthly Mobile Asthma Control Questionnaire (MACQ) in Q5 was created based on the Asthma Control Test and *assessment of asthma symptom control* section in the Global Initiative for Asthma guidelines [24-26]. The MACQ survey is a patient-completed questionnaire with 5 items that assess daytime asthma symptoms, nocturnal symptoms, activity limitations due to asthma, use of rescue medications, and asthma attack (Multimedia Appendices 1 and 2). A total of 4 items included 3 response options, corresponding to a 3-point system, and 1 item for asthma attack included 2 response options, as indicated in Multimedia Appendix 2. The score to be validated in this study is called the MACQ score, which was computed by summing all these 5 items to yield a score ranging from 5 to 15. An MACQ score of 15, a score of 13 or 14, and a score of less than 13 were defined as total control, well control, and poor control of asthma, respectively.

### Adherence Starts With Knowledge 12 (ASK-12)

The Adherence Starts with Knowledge 12 (ASK-12) questionnaire for Japanese adults with asthma was used for assessing adherence to asthma medication in our app [10,27,28]. For each item of the ASK-12 questionnaire, participants were asked to rate their potential barriers to medication adherence using a 5-point scale (all items are listed in Multimedia Appendix 1). For items 1-3 and 8-12, higher scores indicated stronger barriers to adherence. Items 4-7 were reverse-scored so that their final scores were in the same direction as those of the other 8 items. The ASK-12 total score was computed by summing all 12 items. The total barrier count (TBC) was developed as a simplified scoring approach [27,29]. To compute the TBC, the response to each item was dichotomized, with some sections (responses) in the 1-5 scale indicating no barrier “0”, and the remaining sections (responses) indicating a barrier “1” [27]. The ASK-12 questionnaire total score and the TBC score have a possible range of 12-60 and 0-12, respectively, with higher scores representing stronger barriers to adherence.

### Statistical Analysis

Sample normality was examined using the D’Agostino–Pearson test. Differences in parameters between populations were analyzed for significance using the Wilcoxon rank-sum test. To assess correlations between variables, Spearman rank correlation coefficient was used, where appropriate. Differences were statistically significant when *P* values were .05 or less. Statistical analyses were performed in Graph Pad Prism software (version 6; GraphPad Software, Inc.).

## Results

### Clinical and Demographic Characteristics

A total of 1624 patients with physician-diagnosed asthma and 120 patients with physician-diagnosed CVA were enrolled in this study. The baseline characteristics of eligible patients are summarized in Table 1 and the geographic distribution of the study participants is shown in Figure 1B (n=1440). The male-to-female ratio was 38.7:61.3, the median age was 33 years (range 20-74 years), and the median duration of asthma was 15 years (range 0-57 years). The age distribution demonstrated that the study participants were predominantly a younger population (Figure 1C). As much as 91.89% (1598/1739) of individuals remained after exclusion of those who did not respond definitively to the question related to atopy (ie, these individuals selected “Not sure”) and these individuals had some form of atopic predisposition (Table 1 and Multimedia Appendix 1). The familial predisposition to atopic disorders included asthma, allergic rhinitis, atopic dermatitis, urticaria, hay fever, food allergy, and drug allergy (Table 1 and Multimedia Appendix 3) and 88.30% (1532/1735) of participants had some form of familial atopic predisposition (Table 1 and Multimedia Appendix 1).

The patients’ clinical characteristics (Multimedia Appendix 4) showed that 50.97% (889/1744) and 33.03% (576/1744) of the 1744 participants had unscheduled visits and were absent from school or work for asthma symptoms, respectively, even though 62.84% (1096/1744) of participants attended regularly scheduled visits. Overall, 99.43% (1734/1744) of participants reported that their asthma symptoms could be stimulated by a trigger, including house dust, common cold, cold air, and so on (Figure 1D). Moreover, Table 1 shows that 23.14% (402/1737) and 40.75% (705/1730) of participants were current smokers and had pets, respectively. These data suggest that the Zensoku-Log users were patients with poorly controlled asthma which resulted from exposure to environmental factors.

**Table 1:** Demographic characteristics of Zensoku-Log users.

Characteristics		Values
Diagnosis of asthma, n/N (%)		1624/1744 (93.12)
Diagnosis of cough variant asthma, n/N (%)		120/1744 (6.88)
**Sex**		
	Male, n/N (%)	675/1744 (38.70)
	Female, n/N (%)	1069/1744 (61.30)
Age (year), median (range)^a^		33 (20-74)
Body height (m), median (range)^a^		162 (140-196)
Body weight (kg), median (range)^a^		60 (32-135)
BMI (kg/m^2^), median (range)^a^		22.5 (12.7-49.9)
Age at asthma onset (year), median (range)^b^		15 (0-61)
Duration of asthma (year), median (range)^c^		15 (0-57)
Number of remissions of asthma symptoms, n/N (%)		571/1580 (36.14)
Duration in remission (year), median (range)^d^		8 (1-43)
**Smoking history status (never/ex/current)**		
	Never, n/N (%)	955/1737 (54.98)
	Ex-smoker, n/N (%)	380/1737 (21.88)
	Current, n/N (%)	402/1737 (23.14)
Smoking history (pack-year), median (range)^e^		7.5 (0.1-105)
Having animals or birds as pets, n/N (%)		705/1730 (40.75)
Atopic predisposition, n/N (%)		1598/1739 (91.89)
Familial atopic predisposition, n/N (%)		1532/1735 (88.30)

^a^N=1737.

^b^N=1682.

^c^N=1675.

^d^N=406.

^e^N=750.

### Comorbidity

Our data demonstrated that 75.29% (1313/1744) of participants with asthma reported that they suffered from allergic rhinitis (Table 2). Patients with allergic rhinitis usually also have allergic conjunctivitis, and atopic eczema frequently precedes allergic rhinitis. In this survey, 91.89% (1598/1739) of participants with asthma reported that they have a predisposition to atopic disorders (ie, allergic rhinitis, atopic dermatitis, urticaria, allergic conjunctivitis, hay fever, and certain drug-induced reactions; Tables 1 and 2). Among our participants with asthma, 8.20% (143/1744) and 1.32% (23/1744) had aspirin-exacerbated respiratory disease and allergic bronchopulmonary mycoses, respectively (Table 2). Although over half of participants had some symptoms of gastroesophageal reflux (GER), including heartburn and burping, only 20.30% (354/1744) of participants actually suffered from GER and gastroesophageal reflux disease (GERD; Table 2 and Multimedia Appendix 5). These data suggest that Zensoku-Log users require treatment of their comorbidities.

**Table 2:** Comorbidities (N=1744).

Comorbidities	n (%)
Aspirin-exacerbated respiratory disease	143 (8.20)
Allergic rhinitis	1313 (75.29)
Chronic sinusitis	363 (20.81)
Nasal polyp	98 (5.62)
Atopic dermatitis	554 (31.77)
Urticaria	546 (31.31)
Allergic conjunctivitis	614 (35.21)
Pollen hay fever	1039 (59.58)
Food allergy	520 (29.82)
Drug allergy	307 (17.60)
Allergic bronchopulmonary aspergillosis or allergic bronchopulmonary mycoses	23 (1.32)
Chronic obstructive pulmonary disease or emphysema	31 (1.78)
Bronchial ectasia	65 (3.73)
Gastroesophageal reflux disease or gastroesophageal reflux	354 (20.30)
Sleep apnea syndrome	255 (14.62)
Depression or autism	277 (15.88)
History of pneumonia	507 (29.07)
History of tuberculosis	21 (1.20)
Interstitial pneumoniae	18 (1.03)
Lung cancer	1 (0.06)

### Asthma Medications

In the asthma treatment section, 1735 participants completed the questionnaire. A daily inhaled corticosteroid (ICS) had been prescribed as asthma controller medicine for 89.45% (1552/1735) of participants and for all excluded individuals (n=1552) that did not respond to this questionnaire (Table 3). Although a daily oral corticosteroid had been prescribed for 22.07% (383/1735) of participants, a leukotriene receptor antagonist, theophylline, and a long-acting muscarinic antagonist had been prescribed for only 30.66% (532/1735), 15.91% (276/1735), and 4.38% (76/1735) of the participants, respectively (Table 3). These results, taken together with demographic and clinical characteristics shown in Table 1 and Multimedia Appendix 4, suggest that Zensoku-Log users were patients with poorly controlled asthma who require more treatment for asthma control.

**Table 3:** Asthma treatments (N=1735).

Treatment	Value
Oral corticosteroids, n (%)	383 (22.07)
ICS^a^, n (%)	1552 (89.45)
Daily dose of ICS (FP^b^ equivalent dose, µg), median (range)^c^	500 (50-2500)
ICS/LABA^d^ combination products, n (%)	1298 (74.81)
Single-agent ICS products, n (%)	293 (16.89)
Tulobuterol tape, n (%)	320 (18.44)
Leukotriene receptor antagonist, n (%)	532 (30.66)
Theophylline, n (%)	276 (15.91)
Long-acting muscarinic antagonist, n (%)	76 (4.38)
Disodium cromoglycate, n (%)	78 (4.50)
Omalizumab, n (%)	26 (1.50)

^a^ICS: inhaled corticosteroid.

^b^FP: fluticasone propionate.

^c^N=1216.

^d^LABA: long-acting β adrenoceptor agonist.

### Asthma Control

A total of 1727 participants initially answered the Q5 section (asthma control section) of the questionnaire; of these, 1159 (67.11%) participants reported that they experienced good asthma control (Multimedia Appendix 6). However, over half of participants had some symptoms related to asthma or rhinitis and 58.48% (1010/1727) of participants had poor asthma control during the 4 weeks before scoring the MACQ survey (Figure 1E and Multimedia Appendix 6). Table 4 shows the number of each type of response for each item in the MACQ survey; the left column in Figure 1E demonstrates that 28.26% (488/1727) of participants were under complete and total control (Table 4 and Figure 1E). In the 421 participants who repeated the MACQ survey, the MACQ score for the last round was significantly increased as compared with the first round (*P*=.002). Figure 1E demonstrates that the frequency of participants with total and well control of asthma in the last round of the MACQ was increased as compared with the initial round. These results suggest that most Zensoku-Log users were patients with poorly controlled asthma, and that repeated use of the Zensoku-Log app may contribute to achieving better asthma control among users of the app.

**Table 4:** Monthly Mobile Asthma Control Questionnaire (N=1727).

Question	Yes, n (%)		No, n (%)
	Two or more times a week	Once a week or less	
1. Have you had any daytime asthma symptoms in the past 4 weeks?	545 (31.56)	495 (28.66)	687 (39.78)
2. Have you had any asthma symptoms at nighttime during the past 4 weeks?	475 (27.50)	409 (23.68)	843 (48.81)
3. Have you limited your activities, including exercising, because of your asthma in the past 4 weeks?	217 (12.57)	205 (11.87)	1305 (75.56)
4. Have you used your rescue medication to relieve asthma symptoms in the past 4 weeks?	376 (21.77)	342 (19.80)	1009 (58.43)
5. Have you had an asthma attack in the past 4 weeks?	827 (47.89)	—^a^	900 (52.11)

^a^Only yes or no.

### Adherence to Asthma Medication

A previous study reported that patients reporting low adherence to ICS had poorer asthma control than better adherers [30]. In our survey, 61.53% (966/1570) of participants answered that they have stopped taking ICS because 81.1% (784/967) of them had felt better or 81.6% (787/965) of them had improved asthma attacks (Multimedia Appendix 7). Takemura et al. [28] reported that the optimal cut-off value of the ASK-12 total score for discriminating nonadherent patients was 23. The ASK-12 total score and the TBC score in 1569 participants were 28.7 and 4.2, respectively (Multimedia Appendix 8), suggesting that Zensoku-Log users were patients with poor adherence. Among the 306 participants who repeated the ASK-12 questionnaire, the TBC score, but not the ASK-12 total score, was significantly decreased (*P*=.001) in the last round as compared with the first round of answering ASK-12 (Table 5 and Multimedia Appendix 8). Although the scores of Q1 (*P*<.001), Q2 (*P*<.001), Q6 (*P*=.01), and Q7 (*P*=.003) in ASK-12 in the last round of answering were significantly decreased as compared with the initial round, the scores of Q8 (*P*=.03), Q9 (*P*<.001), Q11 (*P*<.001), and Q12 (*P*=.004) of the ASK-12 in the last round of answering were significantly increased as compared with the initial round (Multimedia Appendix 8). These results, nevertheless, suggest that repeated use of the Zensoku-Log app may contribute to reducing medication adherence barriers.

**Table 5:** A summary of the ASK-12 questionnaire (N=306).^a^

Question	TBC^b^, Mean (SD)		*P* value^c^
	First	Last	
1. I just forget to take my medicines some of the time.	0.56 (0.50)	0.43 (0.50)	<.001
2. I run out of my medicine because I don’t get refills on time.	0.49 (0.50)	0.40 (0.49)	.002
3. Taking medicines more than once a day is inconvenient.	0.54 (0.05)	0.51 (0.50)	.28
4. I feel confident that each one of my medicines will help me.	0.17 (0.37)	0.17 (0.37)	>.99
5. I know if I am reaching my health goals.	0.49 (0.50)	0.47 (0.50)	.49
6. I have someone who I can call with questions about my medicines.	0.16 (0.37)	0.11 (0.32)	.04
7. My doctor/nurse and I work together to make decisions.	0.32 (0.47)	0.25 (0.47)	.03
8. Taken a medicine more or less often than prescribed?	0.41 (0.49)	0.45 (0.50)	.23
9. Skipped or stopped taking a medicine because you didn’t think it was working?	0.12 (0.32)	0.14 (0.35)	.30
10. Skipped or stopped taking medicine because it made you feel bad?	0.07 (0.25)	0.08 (0.27)	.59
11. Skipped, stopped, not refilled, or taken less medicine because of the cost?	0.10 (0.31)	0.15 (0.35)	.07
12. Not had medicine with you when it was time to take it?	0.16 (0.37)	0.24 (0.43)	.006
Total	3.59 (2.32)	3.17 (2.40)	.001

^a^We used the Japanese version of ASK-12 in this study.

^b^TBC: total barrier count.

^c^Comparisons performed by Wilcoxon signed-rank test.

## Discussion

### Principal Results

This observational study utilized an mHealth app to survey Japanese patients with asthma and CVA in a real-world setting. To the best of our knowledge, such a study has not been performed previously. This clinical observational study demonstrated that the Zensoku-Log app was useful for remote recruitment of Japanese patients, using an electronic informed consent form, with a wide geographic distribution across Japan, via their iPhones and without requiring intervention by medical staff. This app was more efficient than traditional survey studies that used conventional postal mail, telephone, and web page-based surveys, which are both costly and time-consuming. Different proportions of app downloads were investigated in previous mHealth studies conducted in different parts of the world; for example, in the United States, in the Asthma Mobile Health Study for asthma patients, 19% of downloads were investigated, whereas in the mPower study for Parkinson disease, 20% of downloads were analyzed. In Japan, the DryEyeRhythm study for patients with dry eye evaluated 23% of downloads and the GlucoNote study for patients with type 2 diabetes analyzed 31% of downloads [17,21,31,32]. Although our app was downloaded 7855 times, given the relative ease of app downloading, we attributed the moderate enrollment rate (22.20% [1744/7855] of all downloads), with some drop-off, to the huge volume of questionnaires to be filled in. This app allowed collection of a range of asthma-related data, including symptoms, comorbidities, quality of life, medications, asthma control, and adherence. These data suggested that the Zensoku-Log users were patients with poorly controlled asthma who require more treatment for asthma and their comorbidities, and who need to improve adherence. The geographic distribution of participants (Figure 1B) suggested that patients in metropolitan cities may have higher medical literacy or poorer asthma control or may more frequently be iPhone users. Furthermore, Figure 1C demonstrated that the age distribution in this study was biased toward a younger population. This age distribution may be the cause of the poor asthma control in the real-world setting, suggesting that the mHealth app may reach participants that are typically not reached adequately by traditional studies. Prevalence of allergic rhinitis in the United States is about 15%, based on physician diagnosis, and reaches 30% based on self-reported symptoms [33]. Over 42% of patients with asthma have allergic rhinitis, and up to 40% of patients with allergic rhinitis have or will have asthma [33,34]. Among the Zensoku-Log users, 91.89% (1598/1739) of participants with asthma reported that they have a predisposition to atopic predisposition. These findings and the finding of fewer patients with chronic obstructive pulmonary disease were also associated with a younger population bias in this study as compared with a previous review of comorbidities in severe asthma [35]. Moreover, because the number of patients with GER and GERD was less than the number expected from the results in Multimedia Appendix 5, it is possible that patients themselves underestimate GER. It may be considered that poor asthma control is due to inadequate asthma treatment, poor adherence, inadequate environmental management (eg, current smoking), and inadequate management of comorbidities. Therefore, for asthma management, further education is necessary, including guidelines and development and implementation of a mobile app that can improve a patient’s self-management [36].

Three previous systematic reviews showed that mHealth app-based interventions, including providing reminders, have the potential to improve asthma control and medication adherence in patients with asthma [37-39]. Therefore, it is expected that the app-based intervention with the reminder will be more effective, such as facilitating the delivery of care and connecting patients to their medical staff. The COVID-19 (coronavirus disease 2019) pandemic requires the transformation of traditional medical practices. New digital health innovations will help optimize the efficiency of health care systems and improve patient outcomes. In addition to smartphone therapeutic apps, other components such as sensors, video, social media platforms, wearables, or a combination of these can help enable health care delivery and overcome distance, location, and time constraints. Digital treatment is evolving and it is necessary to involve government authorities/institutions in the approval process. In this study, the repeated use of this app appeared to contribute to achieving better asthma control and to reducing medication adherence barriers (Figure 1E and Table 5). It suggested that even an app designed for observational research, such as ours, which does not involve any app-based interventions, may be a useful educational medium. However, the findings based on the self-reported clinical data in this study may not be representative of the general asthma population and consequently, there is a need for further studies in this area.

### Limitations

Our study had several limitations. First, only Japanese iPhone users could be enrolled, increasing the risk of selection and socioeconomic bias, because it has been shown in the United States that iPhone owners have higher education and income levels than users of other smartphones [17]. However, it is uncertain whether this applies to Japanese users, because according to a StatCounter report [20], the share of iPhones in the Japanese smartphone market is around 60%. However, the proportion of people with low education and low income levels among this group currently remains unknown. Moreover, this study has a potential selection bias toward a younger population. Although app developers should pay attention to usability aspects of mHealth apps during development and release [40], this study paid little attention to these aspects for users of other smartphones and users unfamiliar with using such apps. These biases also present vast differences in the ability of patients to find the health information they need on the internet, a phenomenon also known as “digital divide,” which is one of the most important issues related to the use of mHealth technology. To reduce digital divide across populations disproportionately impacted by health information, it is necessary to make efforts to refine mHealth apps as a study tool (eg, by implementing usability testing to establish how well the app works and serves its intended purpose). All data analyzed in this study were entirely based on participants’ self-motivation and self-reporting, with no way to validate these data by medical personnel; however, such a subjective self-reporting can be prone to bias, with patients likely to overestimate or underestimate the actual scenario [23]. Because these biases are also reported by similar studies using mobile apps, a randomized controlled trial should be performed to confirm the findings of this study. In addition, the validity of the study data collected within the app was difficult to be determined objectively. Our survey questionnaire is developed solely for this app, and thus may limit comparison with studies using standardized instruments. However, the validity of the MACQ score was supported by concordance between this score at baseline and another questionnaire for assessing asthma control. For example, participants’ daily symptoms were all found to be significantly associated with the MACQ score (Multimedia Appendix 9). Moreover, the MACQ score correlated with the TBC score and medication adherence barriers (Multimedia Appendix 9).

Although we requested participants to enter the values of fractional exhaled nitric oxide and peak expiratory flow as objective parameters (Q2 and Q5), these data were missing from their profiles, possibly because participants were unfamiliar with these terms and this information was self-reported (ie, without any assistance of a medical staff). The ResearchKit platform offers the positioning advantage of completely remote recruitment and enrollment, but lack of human communication may cause less motivation for participants to continue with the questionnaire, as compared with studies conducted face to face [17-19,31]. In this study, the proportion of participants repeating the MACQ survey was only 24.38% (421/1727 participants). Therefore, further studies are required to improve retention rates with long-term use. Moreover, although mHealth offers some advantages over traditional face-to-face methods, data collection and interview for delivering behavioral interventions in face-to-face methods are more precise and accurate than the mHealth method. Besides, the face-to-face methods also report better treatment adherence than studies using the mHealth methods.

### Conclusions

We have reported on the use of an mHealth app for Japanese patients with asthma and CVA in a real-world setting. To the best of our knowledge, such a study has not been performed previously. These app users were patients with poorly controlled asthma, who may need more treatment for asthma and their comorbidities. Moreover, repeated use of this app may have improved asthma control. This new mHealth app has novel possibilities for data collection and can reach participants that traditional studies may fail to represent adequately. However, it will be necessary to address the challenges associated with this technology, including selection bias, potential reporting bias, data security, usability issues, digital divides, and low user-retention rate [17].

## Appendix

Multimedia Appendix 1 Survey Questionnaire.

Multimedia Appendix 2 Monthly mobile asthma control questionnaire (MACQ).

Multimedia Appendix 3 Family history of atopy.

Multimedia Appendix 4 Clinical characteristics of Zensoku-Log users.

Multimedia Appendix 5 Questionnaire for the symptoms of gastroesophageal reflux.

Multimedia Appendix 6 Questionnaire for assessing asthma control.

Multimedia Appendix 7 Questionnaire for assessing adherence to ICS.

Multimedia Appendix 8 Summary of ASK-12 questionnaire.

Multimedia Appendix 9 Correlation coefficients for the association of the MACQ score and another questionnaire for assessing asthma control.

## Abbreviations

ASK-12: Adherence Starts with Knowledge-12
CVA: cough variant asthma
FP: fluticasone propionate
GER: gastroesophageal reflux
GERD: gastroesophageal reflux disease
ICS: inhaled corticosteroid
LABA: long-acting β-adrenoceptor agonist
MACQ: monthly Mobile Asthma Control Questionnaire
mHealth: mobile health
TBC: total barrier count
